# Structure and Stoichiometry of the Ton Molecular Motor

**DOI:** 10.3390/ijms21020375

**Published:** 2020-01-07

**Authors:** Herve Celia, Nicholas Noinaj, Susan K Buchanan

**Affiliations:** 1 National Institute of Diabetes and Digestive and Kidney Diseases, Bethesda, MD 20892, USA; herve.celia@nih.gov; 2 Markey Center for Structural Biology, Department of Biological Sciences, and the Purdue Institute of Inflammation, Immunology and Infectious Disease, Purdue University, West Lafayette, IN 47907, USA; nnoinaj@purdue.edu

**Keywords:** Ton complex, TonB, energy-transduction, Gram-negative bacteria, ExbB, ExbD, molecular motors

## Abstract

The Ton complex is a molecular motor that uses the proton gradient at the inner membrane of Gram-negative bacteria to generate force and movement, which are transmitted to transporters at the outer membrane, allowing the entry of nutrients into the periplasmic space. Despite decades of investigation and the recent flurry of structures being reported by X-ray crystallography and cryoEM, the mode of action of the Ton molecular motor has remained elusive, and the precise stoichiometry of its subunits is still a matter of debate. This review summarizes the latest findings on the Ton system by presenting the recently reported structures and related reports on the stoichiometry of the fully assembled complex.

## 1. Introduction

In addition to the cytoplasmic membrane, Gram-negative bacteria possess an additional outer membrane that acts as an efficient barrier against the environment [1]. While small molecules are able to diffuse from the exterior of the cell into the periplasmic space through specialized porins, some nutrients only exist in low concentration in the extracellular medium and hence, need active transport for their uptake [2,3]. However, both the outer membrane and the periplasmic space are depleted in chemical energy sources such as nucleotide hydrolysis, therefore, the energy must come from other sources [4].

**Figure 1:**
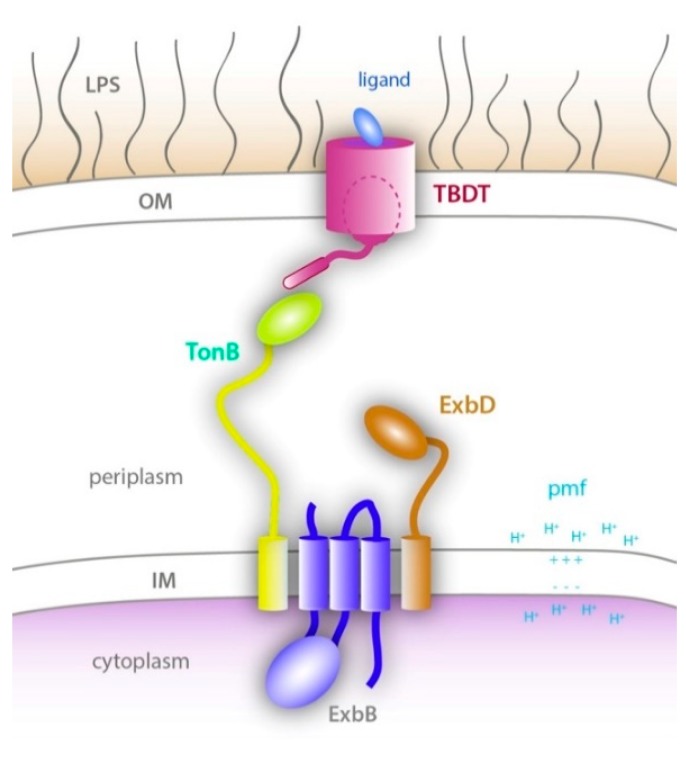
Energy transduction by the Ton system. (adapted from [7]) A schematic of the Ton system which consists of an energy generating and transducing complex located in the inner membrane called the Ton complex (ExbB, ExbD, and TonB), and a TonB-dependent transporter (TBDT) at the outer membrane. Upon ligand binding to the TBDT, TonB interacts with the TonB box of the TBDT. Energy produced by the Ton complex using the pmf is then used to partially unfold or eject the plug domain of the TBDT (dashed oval) to allow ligand import across the outer membrane.

The Ton system is specific to Gram-negative bacteria and allows for the transfer of energy from the inner membrane via the Ton complex to receptors found within the outer membrane [2]. The Ton complex is a unique inner membrane complex that is powered by the proton motive force (pmf) at the inner membrane, much like the ATP synthase [5]. It consists of three integral membrane proteins: TonB, ExbB and ExbD [6]. ExbB and ExbD associate to form the proton translocation part of the motor while the energy derived from the pmf is propagated through the elongated TonB subunit that physically interacts with TonB-dependent transporters (TBDTs) at the outer membrane. This interaction then opens a gate through the barrel domain of the TBDT to allow the entry of the bound nutrient into the periplasm (Figure 1).

The Ton system was first characterized for its role in the uptake of phages T1 and T5 [8] and subsequently studied for its role in iron-bound siderophore import, however, a growing number of different ligands has been identified, from individual zinc ions to polysaccharides [9,10,11,12,13,14]. Bacteriocins, which are able to kill the infected cell with very high efficiency, hijack the Ton system to mediate binding at the surface and to provide energy for their uptake across the outer membrane [15,16,17]. While the mechanism of entry of these bacteriocins into the periplasm is still not well understood, it has been shown in some cases that the bacteriocin uses the same transport pathway through the TBDT as the cognate siderophore [18].

The structures of a large number of TBDTs have been solved by X-ray crystallography. They all show a similar architecture, consisting of a 22-stranded ß-barrel domain filled with a conserved N-terminal plug domain that occludes the pore of the barrel [2]. Upon ligand binding, conformational changes lead to periplasmic exposure of the TonB box, a conserved stretch of amino acids at the N-terminus of the TBDT that has a high affinity for the C-terminal domain of TonB [19]. The exposed TonB box then interacts with the C-terminal domain of TonB [20,21,22,23]. What happens next is largely unknown, but it is generally accepted that the energy derived from the pmf at the inner membrane is propagated through TonB, thereby exerting a force that alters the conformation of the TBDT plug domain which allows the bound ligand to translocate across the outer membrane into the periplasm [20,22,24]. Once the ligand is imported, the plug domain presumably returns to a resting state which leads to the dissociation of TonB.

Unlike for TBDTs where nearly 100 structures have been reported in the Protein Data Bank, structural information for components of the Ton complex has been limited until recently (Table 1). The Ton system was discovered in the 1970s [25,26], but it took a few decades before the isolation and purification of the full complex or subcomplexes was reported [27,28,29]. The structures of soluble periplasmic domains of TonB were first solved by X-ray crystallography and then NMR, showing a dimeric organization, however, this was found to depend on the length of the construct as longer constructs were monomeric [30,31,32,33,34,35,36]. The crystal structures of two TBDTs, BtuB and FhuA, bound to the C-terminal domain of TonB showed that a monomer of TonB associates with the TBDT [20,22]. Shortly after these reports, the NMR structure of the periplasmic domain of ExbD was also reported as a monomer [36]. ExbB was shown to form stable oligomers on its own [28], but it could also be copurified with ExbD [27] and with both ExbD and TonB [7,29]. Surprisingly, the reported biochemical and biophysical characterization of the isolated complexes showed a large range of possible stoichiometries for the different components: 4 ExbB and 2 ExbD [37]; 5 ExbB and 2 ExbD [7]; 6 ExbB and 3 ExbD [38]; 4 ExbB, 1 ExbD, and 1 TonB [29]; 6 ExbB and 1 ExbD or 5 ExbB and 1 ExbD [27]; and 7 ExbB1, 2 ExbD, and 1 TonB [39]. Even with several structures of ExbB and the ExbB-ExbD subcomplexes being recently reported, the stoichiometry of the Ton complex still remains inconclusive. In this review, we will examine what is currently known about the stoichiometries of each component of the Ton complex and discuss future studies needed to not only decipher the true structural state of the Ton complex, but also the mechanism for how it functions.

## 2. TonB—The Energy Conduit of the Ton System

The TonB and ExbD components have a similar topology: a short N-terminal domain, followed by an α-helical transmembrane (TM) domain, a flexible linker, and a C-terminal periplasmic domain (Figure 1). However, the periplasmic domain of TonB is more elongated and able to span the periplasm [54]. The linker between the TM α-helix and the folded C-terminal domain of TonB contains an extended proline-rich region, although the proline-rich nature of the linker does not appear to be essential for TonB activity [55,56]. The structures of different construct lengths of the C-terminal folded domain have been reported, either as dimers with different dimerization interfaces [30,32,33] or as monomers [31,34,35]. While there is some evidence that TonB dimerizes in vivo, the possible role of the observed dimeric structures in the Ton system is still a topic of debate [57,58,59]. Still, the dimer of TonB has been observed in solution and was suggested to play a role in positioning TonB close to the outer membrane through interaction with the peptidoglycan layer [60].

**Figure 2:**
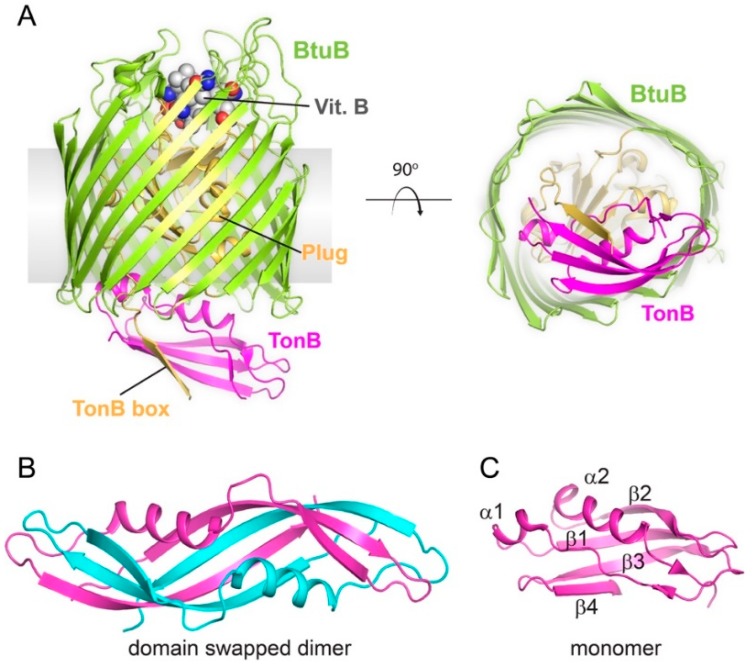
Structures of TonB and TonB-dependent transporters. (**A**) The X-ray structure of BtuB (green), a prototypical TonB-dependent transporter, in complex with vitamin B (spheres) and the C-terminal domain of TonB (magenta) (PBD ID 2GSK). The plug domain is shown in gold. The right panel is a view from the periplasmic face. (**B**) The X-ray structure of the TonB dimer (PDB ID 1IHR) with each monomer in different colors. (**C**) The NMR structure of the TonB monomer (PDB ID 1XX3).

The reported structures of the C-terminal domain of TonB share a common 3-stranded antiparallel β-sheet bundled with two α-helices with an α_1_β_1_β_2_α_2_β_3_ topology (Figure 2) [31,34,35,41,61,62]. TonB has an additional short β-strand at its C-terminus (β_4_), which was found to mediate dimerization in the crystal structures or interact with β_3_ to form a 4-stranded antiparallel β-sheet in the NMR structure of the monomer [30,33,35]. In the BtuB:TonB, FhuA:TonB and FoxA-TonB crystal structures, TonB is a monomer and strand β_4_ is disordered, while β_3_ forms a parallel β-strand interaction with the TonB box of the TBDT (Figure 2A) [20,22,23]. These structural studies are supported by other reports observing that monomeric TonB interacts with the TBDT, which aligns with the fact that TBDTs only have a single TonB box each [21,33,35,60].

## 3. The Structure and Stoichiometry of ExbD within the Ton Complex

**Figure 3:**
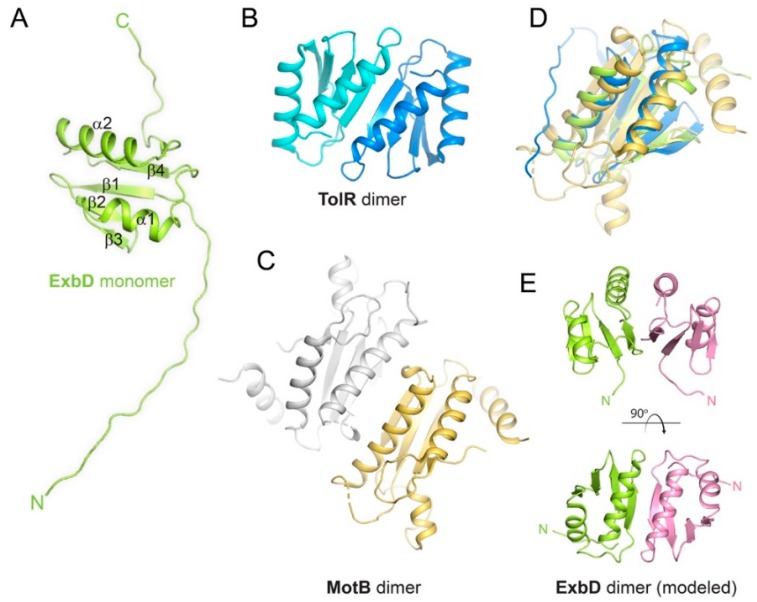
Structures of ExbD, TolR and MotB. (**A**) The NMR structure of monomeric ExbD (PDB ID 2PFU). (**B**) The NMR structure of dimeric TolR (PDB ID 2JWK). (**C**) The X-ray structure of dimeric MotB (PDB ID 3CYP). (**D**) A structural alignment of ExbD (green), TolR (blue), and MotB (gold) depicting the conserved core fold. (**E**) A structural model for the dimer form of ExbD from panel A based on the TolR dimer in panel B.

**Figure 4:**
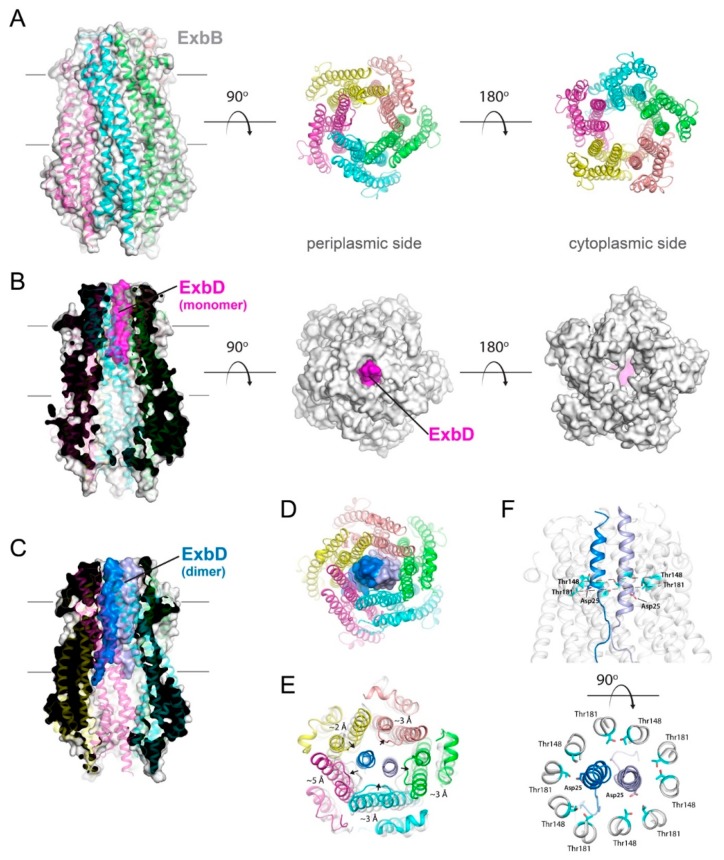
Structures of the pentameric ExbB/D subcomplex. (**A**) The X-ray structure of the ExbB/ExbD subcomplex (PDB ID 5SV0) shown in ribbon and surface. Here, ExbB is a pentamer which has pseudo 5-fold symmetry on the periplasmic side and 5-fold symmetry on the cytoplasmic side. In this structure, solved at neutral pH, ExbD was mostly disordered and missing in the electron density. A cryoEM structure of the pentameric form of ExbB was also recently reported (PDB ID 5ZFV). (**B**) The X-ray structure of the ExbB/ExbD subcomplex (PDB ID 5SV1) at low pH. In this structure, the transmembrane helix for ExbD (magenta) was observed inside the pore of the ExbB pentamer and offset from the plane of the membrane, as shown in the cutaway representation on the left panel. (**C**) The cryoEM structure (PDB ID 6TYI) showing a dimeric form of ExbD sitting within the pentameric pore of ExbB. (**D**) A view of the cryoEM structure from panel C looking down the pore from the periplasmic side. The ExbD dimer is in surface with each chain in a different shade of blue. (**E**) The same view as in panel D, but depicting the conformational changes observed in ExbB to accommodate the ExbD dimer. The structure from panel A is shown in gray for comparison and the subunit shifts are indicated by the black arrows and approximate measurements noted. (**F**) The cryoEM structure from panel C showing the locations of the conserved residues in ExbB (Thr148 and Thr181) and ExbD (Asp25) that are proposed to play a role in proton transport from the pmf for energy production. A side view is shown in the top panel while a top down view is shown in the bottom panel.

The NMR structure of a soluble periplasmic construct of E. coli ExbD (PDB ID 2PFU) shows an extended N-terminal tail (residue 43–63), followed by a structured domain (64–133) and a short flexible C-terminal tail (134–141) (Figure 3A) [36]. The structure of the folded domain consists of a 5-stranded β-sheet stacked against two α-helices. While the NMR structure is a monomer, ExbD is thought to be an oligomer within the Ton complex. This was further confirmed recently with the report of the crystal structure of the ExbB-ExbD_deltaperi_ complex (Figure 4) [7]. While the structure showed that the TM domain of ExbD (residue 22–42) is α-helical (PDB ID 5SV1), only one TM domain of ExbD was observed in the TM pore formed by the pentamer of ExbB (Figure 4B) [7]. Crosslinking and EPR experiments, however, showed that the full length ExbB-ExbD complex contains a dimer of ExbD, with the dimerization domain located in the structured C-terminal periplasmic region [7]. Since only one ExbD TM domain was observed in the ExbB pore in the crystal structure and given that the ExbB pore was not wide enough to fit two α-helices, it was hypothesized that the second TM domain of ExbD must be positioned at the periphery of the ExbB pentamer.

However, this model was challenged by the cryoEM structure of the full length ExbB-ExbD subcomplex embedded in lipid nanodiscs [40] (EMD-20583, PDB ID 6TYI). The 3.3 Å resolution cryoEM structure clearly shows two ExbD TM domains in the ExbB pentameric pore (Figure 4C). In this conformation, the ExbD TM α-helices are parallel to each other and the two essential Asp25 residues pointing in opposite directions toward the conserved Thr148 and Thr181 residues that form a polar ring within the ExbB pentameric TM pore (Figure 4F). The periplasmic domains of ExbD are not visible in the cryoEM map, suggesting that these domains are highly mobile. The dimer interface of the ExbD TM α-helices runs from residues Val20 to Ala41, and the cytoplasmic residues Asn12 to Val20 are in an extended conformation.

The Ton complex has become a prototype for other homologous systems such as the Tol (TolA-TolQ-TolR) and Mot (MotA-MotB) systems. TolQ (29% identity to ExbB) and MotA (22% identity to ExbB) are analogous to ExbB, TolR (27% identity to ExbD) and MotB (16% identity to ExbD) are analogous to ExbD, and TolA (20% identity to TonB) is analogous to TonB. Tol and Mot both also use the pmf to generate mechanical energy, either to maintain the double membrane integrity or to drive the motion of the flagellum, respectively. The structures of the soluble periplasmic domains of ExbD, TolR and MotB show a similar fold for the structured region, and TolR and MotB were found as dimers with similar dimerization interfaces (Figure 3) [36,42,52]. Moreover cysteine scanning and disulfide crosslinking experiments for both the Tol and Mot complexes suggest that the TM domains of TolR and MotB may also be dimeric [63,64], and it was thus surprising to find only one TM domain of ExbD in the crystallographic structure of the ExbB-ExbD_deltaperi_ complex [7]. A likely explanation for ExbB copurifying with only one ExbD TM domain in this structure is that the ExbD_deltaperi_ construct lacked the periplasmic domain, which is known to mediate dimerization [7,65].

A trimer of ExbD has also been proposed based on a cryoEM study of the ExbB-ExbD complex in detergent [38]. In this model, three elongated rods proposed to be three ExbD TM domains were observed within an ExbB hexameric pore. However, no side chains are visible at 6.7 Å resolution, making it difficult to ascertain the true identity of these regions of density.

Because of the extensive similarity in sequence, structure, and function of the Ton, Tol and Mot systems, and with in vivo and in vitro evidence that ExbD is dimeric within the ExbB-ExbD subcomplex, the ExbD dimer can be reliably modeled based on the TolR and MotB structures (Figure 3E). This allows us to form a more complete model of what the overall Ton complex may look like once fully assembled, however, more work is needed to confirm this model.

## 4. The Structure and Stoichiometry of ExbB within the Ton Complex

The stoichiometry of the Ton complex has been a topic of debate for decades and remains controversial. The ExbB-ExbD subcomplex has been reported to contain four copies of ExbB and two copies of ExbD, based on models derived from low resolution negative-stain electron microscopy and biophysical characterization [29,37,66]. This reported stoichiometry was based mainly on quantitative Coomassie staining, blue-native PAGE, and SEC-MALS experiments. Despite the variety of biophysical techniques used in these reports, none of them established an unambiguous stoichiometry. Interestingly, in unpublished results based on negative stain electron microscopy studies, a stoichiometry of five copies of ExbB was reported if co-expressed and purified with ExbD, while six copies of ExbB were reported in the absence of ExbD [67], which aligns well with recent studies discussed below.

One possible explanation for the inconsistencies in these studies may be that the ExbB-ExbD-TonB and/or the ExbB-ExbD complexes have different stoichiometries in vivo versus in vitro and the different studies somehow would reflect this diversity. However, mass spectroscopy experiments performed with native membranes of E. coli cells showed that only the pentameric form of ExbB could be detected [68]. This observation suggests that the tetrameric and hexameric forms of ExbB are either not physiological and do not exist in vivo, or exist in such low quantities that they could not be detected with this technique. Nonetheless, since no overexpression was used in this study, the native mass spectrometry results provide strong evidence that the pentameric form of ExbB corresponds to the most populated physiological state.

**Figure 5:**
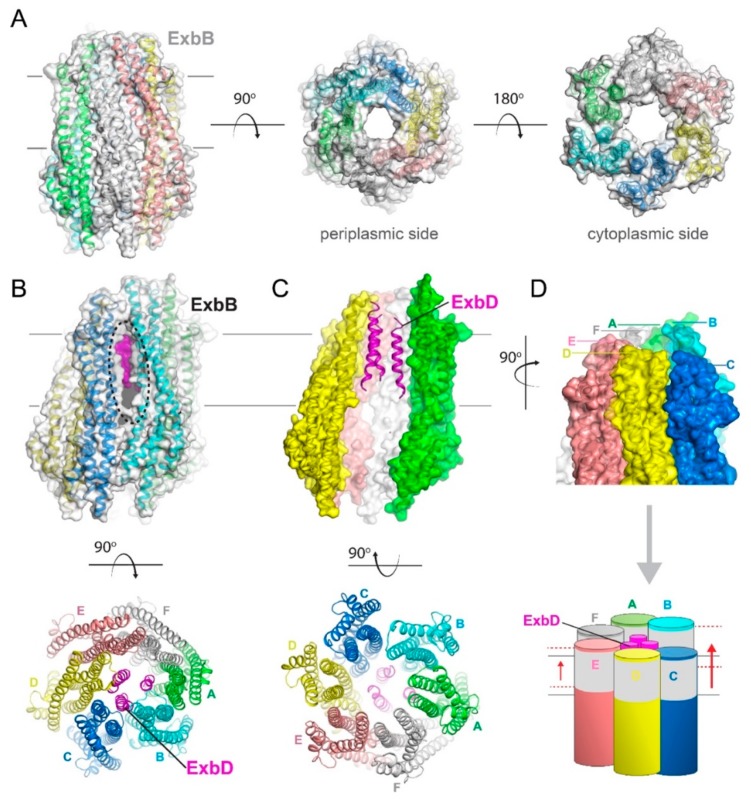
Structures of the hexameric ExbB/D subcomplex. (**A**) The X-ray structure of ExbB (PDB ID 5ZFP) shown in ribbon and surface. Here, ExbB is a hexamer which has 6-fold symmetry on the periplasmic and cytoplasmic sides. (**B**) The cryoEM structure of the ExbB/ExbD subcomplex (PDB ID 5ZFU) having a hexamer of ExbB and trimer of ExbD (magenta). Unlike the structure in panel A, the cryoEM structure has each ExbB monomer offset from the neighboring molecules such that chains B and C differ by ~10 Å in their positions to one another. This offset creates an unusual large portal within the membrane domain between chains B and C, as indicated by the black dashed oval, where ExbD (magenta) is easily observed. Further, due to this unusual arrangement, the membrane girdle of the ExbB hexamer is no longer symmetric, which would presumably induce a slant in the complex within the membrane as depicted in both panel (**C**) (top, chains B and C have been removed for clarity) and panel (**D**), compared to panel (**A**). In panel (**D**) bottom, the membrane domains of each monomer are depicted in gray, while the unusual shift in positions between chains B and C is highlighted by the red dashed lines and red arrow.

Atomic resolution crystal structures of the Ton complex have only recently been reported, with ExbB found as either a pentamer (Figure 4) or a hexamer (Figure 5) [7,38]. The overall fold of the ExbB monomer is similar in both reports, consisting of seven α-helices of varying lengths connected by short loops. For both the pentamer and hexamer forms, a large central cavity is formed, which serves as a direct link between the cytoplasm and periplasm, with the TM domains of ExbD filling the ExbB TM pore region. In both sets of structures, the complex was purified with either a full length or a truncated construct of ExbD [7,38]. Sparse electron density was detected in the ExbB TM pore for both structures, suggesting that the TM domain of ExbD is highly mobile and/or partially dissociated during crystallization. Only for crystals of the pentamer grown at acidic pH was there sufficient density in the ExbB pore to model a single TM domain of ExbD (residues 22–43) (PDB code 5SV1) (Figure 4B) [7].

The cryoEM structure of the ExbB-ExbD complex reconstituted in lipid nanodiscs shows a pentamer of ExbB with a dimer of ExbD TM domains in the pentameric pore with the crystallographic and cryoEM structures of the ExbB pentamers being very similar [7,40]. However, while the ExbB pentamer shows a pseudo 5-fold symmetry in the crystal structure, this symmetry is no longer observed in the cryoEM structure, as the ExbB subunits are extended laterally in the membrane region to accommodate the extra ExbD TM domain.

The crystallographic structures of the Ton subcomplex show that overexpressed and purified ExbB can oligomerize as either a pentamer or hexamer [7,38]. A possible explanation for the hexameric form might be that ExbB alone (i.e., depleted of ExbD) forms a hexamer, as has been previously shown [67]. This would align with the purification strategy used for the reported hexameric structures of ExbB where a histidine-tag was placed on the ExbB subunit only [38], making it difficult to separate excess ExbB-only oligomers from ExbB-ExbD complexes. Since ExbB can form oligomers on its own [28] and is the major component of the ExbB-ExbD complex, it is likely that a mixture of ExbB-only oligomers and ExbB-ExbD complexes were co-purified, which were indeed observed in the statistical analysis of pentamers versus hexamers using cryoEM.

In addition to the hexameric crystal structure, a 6.7 Å cryoEM structure of an ExbB-ExbD complex was determined, having six copies of ExbB and three copies of ExbD (Figure 5B) [38]. While the ExbB subunits in the crystal structure are arranged with 6-fold symmetry within the same plane as the membrane (Figure 5A), surprisingly, in the cryoEM structures, the positions of the ExbB monomers are shifted relative to each other, forming a step-wise spiral perpendicular to the plane of the membrane (Figure 5B–D). This cryoEM structure raises two concerns. The first concern is that the spiral arrangement of the ExbB subunits creates a non-uniform spiraling, hydrophobic girdle along the TM region of the complex, which would not allow uniform, perpendicular positioning within the membrane; rather a tilt would be required for it to rest within the membrane bilayer. The second concern is that the first and last subunits (chains C and B in the Figure 5B–D) of the spiral form are significantly offset from one another by ~10 Å, including the TM domains, such that a large portal has been formed within the membrane region (Figure 5B). Together, these concerns would undoubtedly create stability issues for the Ton complex (as well as the Tol and Mot complexes) and have a profound effect on its role as an energy transducing complex, which has been proposed to utilize rotatory motion within the membrane. Another troubling inconsistency with these studies is that the symmetrical hexamer of ExbB found in the crystal structure was not observed in the cryoEM studies, and the designation of “active” vs. “inactive” states of Ton was not supported by experimental evidence.

The reported structures of ExbB show that ExbB can modulate its lateral contacts to form different oligomeric states (pentamer and hexamer) and can accommodate one or two ExbD TM domains within the pore; with even three TM domains being demonstrated in the hexameric cryoEM structure. The TM domains of ExbD likely undergo conformational changes and/or movement within the ExbB pore when the Ton complex is active in order to generate and transduce energy, and this energy is then propagated through TonB to the outer membrane receptors. Therefore, structural plasticity of the ExbB oligomer is likely a critical factor in order to accommodate the different conformations of the ExbD dimer during energy transduction.

## 5. Mechanism for Energy Transduction by the Ton System

Over the past few decades, several models have been proposed for energy transduction by the Ton complex from the inner membrane to the TBDTs at the outer membrane. The propeller model was based on the observation that the first crystallographic structure of a C-terminal fragment of TonB was dimeric. Here, the dimer of TonB binds to the TonB box of the TBDT and rotates to alter the conformation of the TBDT plug domain, which promotes ligand import [30]. Since then, the propeller model has been challenged by other studies, including the two crystal structures of BtuB and FhuA in complex with TonB, such that there is now convincing evidence that only one monomer of TonB binds to the TBDT [58,60]. Further, the shuttle hypothesis was proposed in which an energized TonB subunit is shuttled between the inner and outer membranes in order to deliver its energy directly to the TBDT, however, this model has since been abandoned [69,70].

The rotational surveillance and energy transfer (ROSET) model is based on in vivo experiments with a N-terminal GFP-TonB construct [71]. Fluorescence polarization measurements have shown that GFP-TonB has a rotary motion in the presence of ExbB, ExbD and the pmf [72,73]. The interpretation of this observation was that after binding to the TBDT, TonB rotates and creates torque, which opens a channel within the TBDT for ligand import. The ROSET model includes a role for the dimer of TonB where one monomer would interact with the peptidoglycan layer in the periplasm, thereby positioning the other monomer closer to the outer membrane where it would interact with the TBDTs [73].

In the pulling model, the C-terminal domain of TonB first binds to the loaded TBDT, and the Ton complex exerts a pulling force that partially unfolds the plug domain to allow the ligand to enter the periplasmic space [74]. Molecular dynamics (MD) simulations of TonB in complex with BtuB have been performed, in which a pulling force is applied on TonB towards the periplasm [24]. The results show that the interaction between the two proteins is strong enough for TonB to remain attached to the TonB box while the applied force gradually unfolds the plug domain. The MD simulations are supported by a number of in vitro experiments including channel measurements of TBDTs in planar lipid membranes showing that the plug domain can be reversibly unfolded with 4M urea [75]. In addition, single-molecule force spectroscopy was used to measure the interaction between the TonB box of BtuB and TonB [76]. In these experiments, the periplasmic domain of TonB was covalently attached to an atomic force microscopy (AFM) probe through its N-terminus and brought into close proximity to an immobilized BtuB. The observed results show that the interaction between TonB and the TonB box of BtuB is strong enough to sustain extension and mechanical unfolding of a portion of the plug domain, before TonB eventually dissociates.

None of these models addresses the way the TonB-ExbB-ExbD complex uses the pmf, and how the generated force is eventually transmitted to the C-terminal domain of TonB. The exact role of ExbB is not well understood, as it does not seem to participate in proton translocation directly [77], although it does contain conserved residues (Thr148 and Thr181) that may be involved. ExbB might act mainly as a scaffolding protein that promotes the assembly of TonB and ExbD [78]. Because of the highly conserved Asp residue in its TM domain, ExbD is likely to play a central role in the proton translocation. The mechanism of proton translocation from the periplasm to the cytoplasmic compartment is largely unknown and the latest cryoEM structure of the ExbB-ExbD complex does not show an obvious proton pathway through the TM pore [40]. ExbD is believed to move during the proton translocation event, possibly through a piston mechanism or a rotation as observed for the F_1_F_0_-ATPase [79], or a combination of both. Regardless of the movement of ExbD, it is unknown how the force and movement are propagated to TonB. The periplasmic C- C-terminal domains of ExbD are likely involved in the process as some interaction between the C-terminal domains of ExbD and TonB has been demonstrated in vivo and found to be dependent on the pmf [65].

Despite the high-resolution structures recently reported using X-ray crystallography and cryoEM, the stoichiometry of the Ton complex and the mechanism for how it generates and transduces energy to the outer membrane remain elusive. Further studies are needed to establish the true composition of the Ton complex to finally put the debate to rest. Is the Ton complex truly composed of two ExbDs within the ExbB pentamer with TonB along the outside? Or, could it be that TonB ends up residing inside the ExbB pore as well? While the hexameric form of ExbB seems like a possible artefact, this observation cannot be excluded; and could the observation of three TM helices loosely demonstrate how two ExbDs and one TonB may reside inside the ExbB pore (as either a pentamer or hexamer)? As such, the structure of the full TonB-ExbB-ExbD complex will give conclusive insight on how TonB interacts with both ExbB and ExbD subunits [7,29] and a structure that includes a TBDT would be the ultimate discovery to put this controversy to rest.

Because of the homology between the Ton, Tol and Mot systems, it is likely that all of these motor proteins have a similar mechanism of action and that they share a similar architecture. The MotA-MotB complex, as well as the PomA-PomB complex, are motor proteins involved in the flagellum rotation and are thought to have a 4-2 stoichiometry [80,81]. The 5-2 stoichiometry of the ExbB-ExbD complex had not been proposed before the X-ray structure of the ExbB-ExbD_deltaperi_ was reported, and it was further supported by the mass spectrometry measurements on native membranes [7,68]. Whether the MotA-MotB, PomA-PomB, and TolQ-TolR complexes share the 5-2 stoichiometry remains to be determined. Some new homologues belonging to the Ton family have been identified recently. The Agl-Glt machinery uses the pmf at the inner membrane and is involved in bacterial gliding motility, and the Poc complex is involved in swimming motility [82,83]. Because these proteins exist only in Gram-negative bacteria, they represent a potential target for the design of new antibiotics. In this respect, a deeper understanding of how some bacteriocins use the Ton uptake pathway to enter and kill the bacterial cell will be helpful. The high specificity and efficiency of these bacteriocins could help to design new drugs that would mimic their mode of action [84,85].

One of the multiple challenges in elucidating how the Ton complex works will be to design an in vitro assay that can test multiple hypotheses and models. Unlike other systems that can be actively reconstituted in a single membrane, the Ton system requires not only a dual membrane, but a proton gradient to power the Ton complex. The purification of a stable Ton complex is a first step [7,29], but mimicking the proton gradient and the ability to measure conformational changes of TonB and/or ExbD will also be required. While crystallizing such a system will be challenging, cryoEM is the method of choice to elucidate the stoichiometry and near atomic resolution details of the whole TonB-ExbB-ExbD complex [86]. CryoEM might also be used to investigate the interaction between Ton and the TBDTs and the conformational changes taking place during the transport cycle. These structural studies would also need to be complemented with other biochemical and biophysical approaches such as crosslinking and EPR/DEER to investigate exactly how the Ton system functions in energy transduction and ligand gating at the outer membrane [87,88].

## Figures and Tables

**Table 1:** Summary of Ton-related structures.

PDB ID	Method; Resolution	Species	ExbB	ExbD	Other	Detergent(s)	Reference
6TYI	CryoEM; 3.3 Å	*E. coli*	5	2		MSP1E3D1 nanodisc	Celia et al., Nat Comm Bio 2019 [40]
5ZFP	X-ray; 2.84 Å	*E. coli*	6	0		0.5% C_8_E_4_ or 0.07% C_10_E_5_	Maki-Yonekura et al., ELife 2018 [38]
5ZFU	CryoEM; 6.7 Å	*E. coli*	6	3		0.002% LMNG	Maki-Yonekura et al., ELife 2018 [38]
5ZFV	CryoEM; 7.1 Å	*E. coli*	5	1		0.002% LMNG	Maki-Yonekura et al., ELife 2018 [38]
5SV0	X-ray; 2.6 Å	*E. coli*	5	1 (NTD)		0.08% C_10_E_5_	Celia et al., Nature 2016 [7]
5SV1	X-ray; 3.5 Å	*E. coli*	5	1 (NTD)		0.08% C_10_E_5_	Celia et al., Nature 2016 [7]
2PFU	NMR	*E. coli*		1 (CTD)			Garcia-Herrero et al., 2007 [36]
2GRX	X-ray; 3.3 Å	*E. coli*			1 (TonB CTD); 1 (FhuA)		Pawelek et al., 2006 [20]
2GSK	X-ray; 2.1 Å	*E. coli*			1 (TonB CTD); 1 (BtuB)		Shultis et al., 2006 [22]
1IHR	X-ray; 1.55 Å	*E. coli*			2 (TonB CTD)		Chang et al., JBC 2001 [30]
1QXX	X-ray; 2.7 Å	*E. coli*			2 (TonB CTD)		Koedding et al., JBC 2004 [33]
1XX3	NMR	*E. coli*			1 (TonB CTD)		Peacock et al., JMB 2005 [35]
1U07	X-ray; 1.13 Å	*E. coli*			1 (TonB CTD)		Koedding et al., JBC 2005 [32]
6FIP	NMR	*P. aeruginosa*			1 (TonB CTD)		Oeemig et al., PeerJ 2018 [34]
5LW8	NMR	*H. pylori*			1 (TonB CTD)		Ciragan et al., 2016 [31]
2K9K	NMR	*V. anguillarum*			1 (TonB CTD)		Lopez et al., Biochem J 2009 [41]
2JWK	NMR	*H. influenzae*			1 (TolR CTD)	-	Parsons et al., 2008 [42]
2JWL	NMR/SAXS	*H. influenzae*			1 (TolR CTD)		Parsons et al., 2008 [42]
5BY4	X-ray; 1.7 Å	*E. coli*			1 (TolR CTD)		Wojdyla et al., 2015 [43]
1S62	NMR	*E. coli*			1 (TolA CTD)		Deprez et al., JMB 2005 [44]
1LR0	X-ray; 1.91 Å	*P. aeruginosa*			1 (TolA CTD)		Witty et al., EMBO J 2002 [45]
2X9A	X-ray; 2.47 Å	*E. coli*			1 (TolA CTD)		Lorenz et al., JMB 2011 [46]
1TOL	X-ray; 1.85 Å	*E. coli*			1 (TolA CTD)		Lubkowski et al., Structure Fold Des 1999 [47]
3QDR	X-ray; 2.65 Å	*E. coli*			1 (TolA CTD)		Li et al., JBC 2012 [48]
3QDP	X-ray; 2.15 Å	*E. coli*			1 (TolA CTD)		Li et al., JBC 2012 [48]
2ZOV	X-ray; 2.0 Å	*S. typhimurium*			2 (MotB)		Kojima et al., Mol Micro 2009 [49]
2ZVY	X-ray; 1.75 Å	*S. typhimurium*			2 (MotB)		Kojima et al., Mol Micro 2009 [49]
2ZVZ	X-ray; 2.4 Å	*S. typhimurium*			2 (MotB)		Kojima et al., Mol Micro 2009 [49]
5Y3Z	X-ray; 2.0 Å	*S. typhimurium*			2 (MotB)		Kojima et al., Mol Micro 2018 [50]
5Y40	X-ray; 2.8 Å	*S. typhimurium*			2 (MotB)		Kojima et al., Mol Micro 2018 [50]
3KHN	X-ray; 2.03 Å	*D. vulgaris*			2 (MotB)		No citation
3S02	X-ray; 2.5 Å	*H. pylori*			2 (MotB)		O’Neill et al., Acta Crys D 2011 [51]
3S0H	X-ray; 2.1 Å	*H. pylori*			2 (MotB)		O’Neill et al., Acta Crys D 2011 [51]
3S0W	X-ray; 2.5 Å	*H. pylori*			2 (MotB)		O’Neill et al., Acta Crys D 2011 [51]
3S0Y	X-ray; 1.8 Å	*H. pylori*			2 (MotB)		O’Neill et al., Acta Crys D 2011 [51]
3S03	X-ray; 2.5 Å	*H. pylori*			2 (MotB)		O’Neill et al., Acta Crys D 2011 [51]
3S06	X-ray; 1.8 Å	*H. pylori*			2 (MotB)		O’Neill et al., Acta Crys D 2011 [51]
3CYP	X-ray; 1.6 Å	*H. pylori*			2 (MotB)		Roujeinikova, PNAS 2008 [52]
3CYQ	X-ray; 2.3 Å	*H. pylori*			2 (MotB)		Roujeinikova, PNAS 2008 [52]
3IMP	X-ray; 2.5 Å	*H. pylori*			2 (MotB)		Reboul et al., Plos one 2011 [53]
